# Understanding Barriers and Facilitators of Maternal Health Care Utilization in Central Myanmar

**DOI:** 10.3390/ijerph17051464

**Published:** 2020-02-25

**Authors:** Maja Aleksandra Milkowska-Shibata, Thin Thin Aye, San Myint Yi, Khin Thein Oo, Kyi Khaing, Marlar Than, Thinzar Win, Su Yi Myo, Su Yi Toe, Heidi Sierra West, Kristin Melissa Ringstad, Lizeth Galarza, Can Meng, Tomoyuki Shibata

**Affiliations:** 1 Global Environmental Health LAB, Brooklyn, NY 11026, USA; milkowska.maja@gmail.com (M.A.M.-S.); hwest@gehlab.org (H.S.W.); kristinringstad@gmail.com (K.M.R.); lizethgalarza@gmail.com (L.G.); 2 Department of International Relation, Yadanabon University, Mandalay 05063, Myanmar; dr.thinnthinnaye01@gmail.com (T.T.A.); sanmyintyi2012@gmail.com (S.M.Y.); 3 Department of Geography, Meiktila University, Meiktila 05181, Myanmar; khintheinoo65@gmail.com; 4 Department of Geography, Mandalar Degree College, Mandalay 05052, Myanmar; marakhaine@gmail.com; 5 Department, of Applied Economics, Meiktila University of Economics, Meiktila 05181, Myanmar; marlarthan007@gmail.com; 6 Department of Economics, Mandalay University, Mandalay 05032, Myanmar; thinzarthinzarwinaung@gmail.com; 7 Mandalay City Development Committee, Mandalay 100102, Myanmar; suyimyo.sym@gmail.com; 8 Department of Preventive and Social Medicine, University of Medicine, Mandalay 05024, Myanmar; suyitoe@gmail.com; 9 Department of Health Policy and Management, University of California, Los Angeles, CA 90095, USA; 10 Yale Center for Analytical Sciences, Yale University, New Haven, CT 06511, USA; can.meng@yale.edu; 11 Public Health Program, Northern Illinois University, Dekalb, IL 60115, USA; 12 Center for Southeast Asian Studies, Northern Illinois University, Dekalb, IL 60115, USA

**Keywords:** maternal health, health care utilization, Myanmar

## Abstract

The study objective was to examine barriers and facilitators of maternal health services utilization in Myanmar with the highest maternal mortality ratio in Southeast Asia. Data for 258 mothers with children under five were extracted from a community health survey administered between 2016 and 2017 in Mandalay, the largest city in central Myanmar, and analyzed for associations between determinants of maternal health care choices and related outcomes. The study showed that late antenatal care was underutilized (41.7%), and antenatal care attendance was significantly associated with geographical setting, household income, education, and access to transportation (*p* ≤ 0.05). Less than one-third of women gave birth at home and 18.5% of them did so without the assistance of traditional birth attendants. Household education level was a significant predictor for home delivery (*p* < 0.01). Utilization of postnatal care services was irregular (47.9%–70.9%) and strongly associated with women’s places of delivery (*p* < 0.01). Efforts geared towards improving maternal health outcomes should focus on supporting traditional birth attendants in their role of facilitating high-quality care and helping women reach traditional health facilities, as well as on maternal health literacy based on culturally appropriate communication.

## 1. Introduction

Reducing maternal mortality and improving maternal health represent global priorities, as illustrated by the Sustainable Development Goals. The global maternal mortality ratio in 2017 was estimated at 211 maternal deaths per 100,000 live births [1]. Higher ratios of death related to pregnancy and childbirth disproportionately affect low-to-middle income countries, where 94% of all maternal deaths occurred in 2017 [1]. Although maternal mortality ration declined by approximately 38% worldwide from 2000 to 2017, considerable efforts are required in low-to-middle income countries, especially in Africa and Asia, to achieve Target 1 of the Sustainable Development Goal 3: Reducing global maternal mortality ratio to less than 70 per 100,000 live births by 2030 [2].

Approximately 75% of all maternal deaths are associated with severe bleeding, infections, high blood pressure during pregnancy, complications from delivery, and unsafe abortions, conditions that are highly preventable [3]. Maternal health interventions, including access to high-quality care during pregnancy, delivery, and after childbirth, are considered highly effective in reducing the burden of maternal mortality and morbidity, particularly in low-resource settings [1,4,5]. Various factors contribute to access to quality maternal health services, from those well-documented, such as poverty, distance to health care facilities, access to information, quality health services, and cultural beliefs and practices [6], to those that have been identified most recently, including green space coverage [7]. However, it is important for local stakeholders to identify which barriers are most prevalent in their communities. 

Compared to maternal health crises in Sub-Saharan Africa (maternal mortality ratio of 542 per 100,000 live births) and Southern Asia (maternal mortality ratio of 157 per 100,000 live births), which accounted for approximately 86% of global maternal deaths in 2017 [1], maternal health care utilization in Southeast Asia has not been widely studied quantitatively. Southeast Asia is home to many of the most economically, socially, and culturally diverse countries in the world [8]. Those differences are likely to create unique challenges in providing maternal health interventions in the region. As of 2017, Myanmar had the highest maternal mortality ratio in Southeast Asia (250 per 100,000 live births), which is almost twice as high as the regional average of 137 deaths per 100,000 live births [1]. In spite of progress following the transition from military rule to a civilian government in 2011, Myanmar, classified as a low-to-middle income country, continues to suffer from numerous challenges, including those in the health care sector [9].

To date, little is known about the patterns of maternal health services utilization in Myanmar. Moreover, large variations in maternal mortality exist between the country’s regions. As of 2014, the maternal mortality ratio of the Mandalay region accounted for 280, which was similar to the union’s average of 282 deaths per 100,000 live births [10]. Such results place the region in-between in terms of the in-country maternal mortality ratio, ranging from 157 in the Tanintharyi State to 357 deaths per 100,000 live births in the Chin State, characterized by high fertility rates. The level of maternal mortality was also higher in rural areas (310 deaths per 100,000 live births) compared to urban communities (193 deaths per 100,000 live births), which highlights the need to further explore the landscape of maternal health services and utilization trends at the local level. Uncovering the determinants of healthcare service utilization plays a crucial role in understanding maternal well-being. Therefore, the objective of this study was to evaluate barriers and facilitators of maternal health care utilization in Myanmar, including (1) antenatal care, (2) delivery, and (3) postnatal care. To address the knowledge gap concerning the mentioned study areas in rural populations, the study findings have been presented according to an urban and rural setting and discussed with the aim of improving evidence-based decision-making on maternal health services and further enhancing maternal health outcomes in Myanmar and also in other similar low-to-middle income countries. 

## 2. Methods

### 2.1. Study Site

To date, there are a limited number of studies addressing challenges in access to maternal health services in Myanmar, particularly in rural areas [11], within ethnically diverse regions [12] and for migrant populations [13,14]. It has been reported that 75.7% of the overall maternal deaths occurred in rural areas, whereas 23.4% in urban settings in 2017 [15]. Therefore, this study was conducted in two townships of Mandalay City (population 1.2 million), the largest city in central Myanmar [16]. One township, Chanayethazan, representing the urban population, lies in the heart of the city, while the other, rural township of Amarapura is located an hour drive from the city center. 

### 2.2. Data Collection

Data collection took place as part of the second workshop and collaborative research for the Sustainable Development Goals in Myanmar approved by the Ministry of Education and directed by Global Environmental Health LAB in Mandalay in 2016. The study protocol was approved by the Northern Illinois University Institutional Review Board (IRB# HS16-0174: Community and occupational health associated with the Sustainable Development Goals). 

The reported research falls under a broader study employing a community and occupational health survey. The research tool was developed by a multidisciplinary team of scholars from Myanmar and the US based on literature reviews, as well as previous studies related to the Millennium Development Goals and child health, water, sanitation, and hygiene (WASH) and environmental and occupational health conducted in Southeast Asia [17,18,19]. The maternal health component of the survey consisted of 26 questions covering participants’ demographics (e.g., age, ethnicity, household income, level of education, and occupation) and various aspects of health services related to antenatal care (e.g., number of attended visits, distance to health facility, obtained services, and experienced complications); delivery (e.g., delivery type, place of delivery, and delivery with skilled birth personnel); and postnatal care (e.g., services obtained within first 24 hours after birth and number of postnatal checkups). It was created based on available national maternal health surveys [20] and other low-to-middle income countries [21], as well as regional reports [13] and recommendations [22]. The questionnaire was validated by local faculty members as to the question choice, comprehensibility, and validity. The survey was further pilot-tested on a small sample to validate its accuracy, cultural appropriateness, and burden on the participants. The questionnaire was developed in English and translated into Burmese, the official language in Myanmar, with the help of local faculty who administered it between June 2016 and September 2017. 

A convenience sampling approach was utilized to identify eligible participants (e.g., local residents at the age of 18 and older) together with a geographic sampling methodology to cover areas in the proximity of river bodies to study other issues under the survey (e.g., water-borne diseases or migration patterns). Face-to-face interviews focusing on most recent pregnancy experiences were conducted in Burmese with women from age eighteen onwards who had children under five years of age. Verbal consent was obtained from participants as in the case of areas characterized by low literacy. The collected responses were translated back to English. A total of 937 adults from the urban township of Chanayethazan (55.5%) or the rural township of Amarapura (44.5%) participated in the survey 

### 2.3. Data Analysis

All data obtained from the survey questionnaires were entered and organized in Microsoft Excel as the 2016–2017 Mandalay Community and Occupational Health (MCOH) dataset (*n* = 937). Relevant data was extracted for 258 female participants who had children under five years of age from this dataset and verified for accuracy prior to statistical analyses. Household poverty level was determined based on the number of people in the household and the income threshold of $1.25 per day per person, according to the Sustainable Development Goal 1, Target 1.1: Eradicate extreme poverty [2]. 

Chi-squire and *t*-tests were used for categorical and numerical variables, respectively, to compare differences in demographics and maternal health services utilization between urban and rural settings. The odds ratio was used to evaluate associations between determinants of maternal health care choices and related outcomes, and logistic regression was used to control possible confounders, including education and household income. Missing values were excluded from all analyses. Statistical significance level was set to 0.05 for detecting differences and associations. All analyses were conducted using IBM SPSS Statistics 25 (IBM, Armonk, NY, USA) and SAS 9.4 (SAS Institute Inc., Cary, NC, USA).

## 3. Results

### 3.1. Study Population

The average age of the study population, mothers with children under five, was 32 ± 8 years old. Those living in the rural setting were approximately three years younger on average than mothers in the urban setting (*p* = 0.02) (Table 1). The women’s education levels were significantly different between urban and rural settings (*p* < 0.01). The median education levels in urban and rural settings were upper secondary school and lower secondary school, respectively, which were significantly different (*p* < 0.01) and higher than the union’s level (where completed primary education was most common), as well the Mandalay State (incomplete primary education). It should be noted that compulsory education in Myanmar includes primary and lower secondary schools. Occupations varied significantly between urban and rural settings (*p* < 0.01), with the most common occupation being housewife (35.9% in the urban and 44.9% in rural setting). Median monthly household income in both settings was Myanmar Kyats 250,000 (an equivalent of $200). More women in the rural setting (35.4%) lived in households below the extreme poverty line of $1.25 per day per person (*p* = 0.02). 

### 3.2. Antenatal Care

The most common choices for maternal health care services were significantly different by setting: governmental (54.6% in the rural setting) or private clinics/hospitals (61.7% in the urban setting) (*p* < 0.01) (Table 2). Only 6.6% of the participants, who all lived in the rural setting, visited traditional birth attendants as the primary maternal health care service providers. The use of traditional birth attendants has been seen only in the rural setting (*p* = 0.02). Women from households where the highest level of education was less than high school were approximately seven times (OR = 6.63; 95% CI 1.43–30.7; *p* = 0.02) more likely to choose traditional birth attendants compared with women from households with high school education and higher. 

Overall, women followed the recommendations on the number of antenatal care visits during each trimester, overutilizing the services during the first and second trimester. However, many women did not make the recommended number of visits during each of the 1st, 2nd, and 3rd trimesters: 16.6%, 26.8%, and 58.3%, respectively. Women in the rural setting were approximately three times (OR = 2.92; 95% CI 1.58–5.38; *p* < 0.01) more likely not to meet the recommended five visits during the third trimester. 

The most frequently mentioned reason for not attending antenatal care was the perception that it was unnecessary (36.8%). Such perceptions showed statistical associations with levels of educations; for example, women with less than primary education were more likely to hold these views (*p* = 0.03). When controlling for primary education and median income, women were approximately 17 times (adjusted odds ratio, or AOR = 17.7; 95% CI 1.72–183; *p* = 0.02) more likely not to visit an antenatal care service provider for the recommended visits if they lived in the rural setting (Table 6). The women who cited lack of transportation as the reason for not seeking antenatal care visits were approximately 14 times (OR = 14.3; 95% CI 1.56–131; *p* = 0.01) more likely not to meet the recommendation of two visits during the second trimester compared to women expressing other reasons.

It took 30 ± 29 min on average for the women to travel to antenatal care service providers, which was significantly longer (*p* < 0.01) than the time needed to visit general health care providers (22 ± 18 min on average). Distance was a reason for not seeking antenatal care only for women in the rural setting (*p* = 0.01). If walking was a primary mode of transportation to attend an antenatal care visit, rural women were approximately nine times (OR = 8.89; 95% CI 1.40–56.6; *p* = 0.02) more likely not to make the recommended number of third trimester visits than urban women. 

The participants who visited clinics, hospitals, and health facilities run by the Myanmar Maternal and Child Welfare Association (MMCWA) largely received most recommended antenatal care services, including physical examination (90.7%), gynecological examination (82.5%), or ultrasound (85.6%). The same services were received by significantly fewer mothers (0%–35.7%) when visiting a traditional birth attendant (*p* < 0.01) (Table 3).

### 3.3. Labor and Delivery

Over a quarter (27.1%) of women delivered their last babies at home, while the majority (72.9%) gave birth in conventional health care facilities (Table 4). Most women (94.7%) delivered with skilled birth attendants but 5.3% did not. While all women who delivered at health care facilities were assisted by skilled birth attendants, 18.5% of the women who delivered at home did so without the assistance of skilled birth attendants. Women reported low rates of delivering a stillborn baby (1.3%) and spontaneous abortions (3.0%) in the past. All reported stillborn baby cases were observed among the women who gave birth at home. Spontaneous abortions were 14.3 times (OR = 14.3; 95% CI 1.63–125; *p* = 0.01) more likely to be observed in women giving birth at home compared to those delivering in conventional health care facilities. 

Although giving birth at home was more common in the rural (34.1%) than urban (6.6%) setting (*p* < 0.01), household education level was a significant predictor for home delivery after controlling for confounding variables. Women from households in which compulsory education was not attained were approximately three times (AOR = 3.31; 95% CI 1.72–6.38; *p* < 0.01) more likely to deliver at home compared to those with compulsory education when controlling for median income. It was assumed that the women who visited conventional antenatal care service providers would give birth at conventional health facilities, while those who visited traditional birth attendants as their antenatal care service providers would give birth at home with their assistance. However, 21% of women who visited conventional health care facilities for antenatal care gave birth at home. Similarly, 18.8% of women who initially visited traditional birth attendants for antenatal care gave birth at conventional health care facilities. The most common reason for not delivering at a health care facility was that women preferred having their babies delivered at home (81.1%). The other reasons included lack of money (9.4%), distance to a health facility (3.8%), and lack of transportation (3.8%). Those reasons were significantly different between urban and rural settings (*p* < 0.01). 

### 3.4. Postnatal Care

The majority of mothers (88.9%) and their babies received postnatal care within 24 hours after giving birth. Over three-quarters (77.6%) of women and infants were visited by skilled birth attendants in the first week after birth. Common postnatal care services received included examinations (e.g., physical examination—79.9%, child growth and weight monitoring—74.1%, and a blood test for anemia—47.9%); maternal education (e.g., information on clinical signs of newborn illness—74.1%, family planning—58.4%, and support for new parents—64.1%); counseling (e.g., breastfeeding—78.6% and postpartum depression—57.5%); and provision of nutritional supplements (65.5%) (Table 5). 

When delivering at home with a traditional birth attendant, mothers were about 38 times (AOR = 38.5; 95% CI 9.17–167; *p* < 0.01) more likely not to receive such postnatal care services when controlling for household compulsory education and median monthly household income. Women who delivered babies at home were also approximately three times (OR = 2.89; 95% CI 1.50–5.54; *p* < 0.01) more likely not to be visited by skilled birth attendants Table 6.

## 4. Discussion

The results of this study show significant variations in the utilization of maternal health services among women in Mandalay, poor use of late antenatal care, low presence of skilled birth attendants during home delivery, and unsatisfactory postnatal care services provision. Burmese women have been reported to attain higher educational levels when compared to men [23]. In 2014, nearly 60% of those who graduated from university and almost two-thirds of those with postgraduate qualifications were women [23]. The main predictors of poor maternal health choices were household education as well as residing in rural areas and delivering at home.

It has been recommended for pregnant women to visit antenatal care providers at least one time during the first trimester, twice during the second trimester, and five times during the third trimester [24]. While reasons for the overall overutilization of antenatal care have not been explored in the study, such behavior could be a result of pregnancy complications or anxiety in women classified as at high risk of such complications [25]. The concerning lack of the perceived value of antenatal care and its association with women’s education, previously identified by [26] in a local study, remains to be an important and long-manifested barrier to the utilization of maternal health services in many low-income settings [27,28]. Coverage of essential antenatal care services received by the women was generally higher compared with available evidence from Myanmar (below 80%) [29]. However, high use of early antenatal care and low antenatal care attendance later during pregnancy is in contrast with other studies [26,30], suggesting an undesirable tendency. 

While lower than the rates of home delivery reported earlier, especially in rural settings [26,29], one-fourth of reported deliveries took place at home and were scarcely accompanied by skilled birth attendants. This is one of the factors hindering progress towards reducing maternal mortality. Home deliveries were largely chosen according to women’s preferences, which is commonly related to the cultural dimensions of pregnancy and birth, manifesting itself by the perception of comfort and privacy associated with home delivery [31] or the development of unique relationships between future mothers and traditional birth attendants in community settings [32]. Traditional birth attendants remain vital providers of maternal and neonatal care whose responsibilities vary in different low-to-middle income countries [33]. Despite increasing access to skilled birth personnel in Myanmar, traditional birth attendants continue to be utilized, especially in rural areas. Besides essential services related to antenatal care, delivery, and postnatal care, they frequently assist with everyday care of infants within the first week following the delivery [34]. In addition, the competences of traditional birth attendants in community settings, especially in conflict areas within the country, have been further enhanced through local networks of service provision, where they are also responsible for overseeing colleagues [35]. In the local context, the important role that traditional birth attendants play in maternal health care provision, including linking women to the formal health system and services provided by skilled birth attendants, is especially relevant. Recognizing that access to skilled birth attendants in both geographic and financial terms remains a major challenge and population sizes are often too large for skilled birth attendants to provide full antenatal and postnatal care coverage [27], traditional birth attendants play a crucial role in first-line care, maternal education, and complementing the formal health system. The discourse on the role of skilled and traditional birth attendants in improving maternal health outcomes in Myanmar needs to be further supplemented by available evidence from other low-to-middle countries [36].

Although overall postnatal care service utilization within 24 hours after birth was much higher compared with other studies from Myanmar [37], postnatal care services were underutilized compared with antenatal care services. Importantly, the likelihood of not receiving such services was much higher for those that delivered at home and had lower education. As highlighted by Mon et al. [38], the preference of home delivery can be largely related to women’s misconception on postnatal care. Based on Burmese customs and traditional beliefs, some women tend to choose strict home confinement for up to a week after delivery, which may hinder their timely and adequate attendance of postnatal care. Moreover, lack of culturally appropriate postnatal care information, pointed out by Diamond-Smith et al. [31], may drive women to resort to traditional practices that sometimes carry harmful effects both for women and their babies. Experiences of delivery and the postnatal period among Burmese women may be further influenced by religious beliefs, as 99.2% of the surveyed women identified themselves as Buddhists, for which it is common to perceive health outcomes as a result of one’s fate [39].

The study has several limitations. The distinction between rural and urban study areas used for the purpose of the analysis may not fully reflect the actual characteristics of the two areas. Specifically, although mostly rural (66%) [40], Amarapura Township owes its origin to the expansion of urban populations of Mandalay City to remote areas, and approximately one-third of its territory can be characterized as an urban environment. Due to the sampling method used for the original database, the study population was distributed as follows: 26.0% of participants lived in the urban, while 73.9% lived in the rural settings. Such a distribution does not adequately represent the actual distribution of the target population in this study site (e.g., 65.4% of women with children under five live in urban Chanayethazan and 34.6% in rural Amarapura, based on a 2014 census, [41]. However, the study population proportions reflected a reported distribution of maternal deaths in Myanmar (23.4% in urban and 75.7% in rural settings) [15]. Thus, it was justified to include larger survey data from women in rural settings in this study. The nature of the survey required that participants were asked to report on past experience with maternal health services and delivery. The survey asked about experiences related to the last pregnancy; however, its design did not allow for data analysis per strata reflecting such dates (e.g., comparing experiences of mothers that delivered a year ago with those that gave birth five years ago). Consequently, asking women to reflect on their experiences from, in some cases, as long as nearly five years prior to the point of survey administration could have generated recall bias, which poses a concern when using self-reported data [42]. Nevertheless, evidence suggests that mothers’ recall of events related to pregnancy remains reliable over extended periods of time [43]. Additional data sources, such as home-based records, may need to be used in similar studies to complement self-reported accounts. Lastly, the nonrandomized sampling technique employed in the study may have resulted in recruiting participants having certain characteristics, as in this case rural communities, for example. 

## 5. Conclusions

Inconsistent utilization of maternal health services throughout pregnancy and the unsatisfactory uptake of skilled birth attendants in community settings provide important evidence for improving policy intervention. First of all, as infrastructural barriers to delivering maternal health care were identified, such as lack of transportation or health infrastructure, especially in rural settings, employing interventions aimed at enhancing the technical capacities of traditional birth attendants should be supported as more feasible in low-income settings (e.g., with the use of mobile technology, such as ultrasounds). Such an approach could ensure continuous skill developments and enhance the role of traditional birth attendants as facilitators of high-quality care in community settings for women who otherwise lack access to skilled birth personnel. Secondly, greater focus should be placed on increasing maternal health literacy (e.g., on the importance of antenatal care, delivery with skilled birth attendants, and postnatal care) for mothers’ and babies’ well-being based on culturally appropriate communication. Future research is needed to understand the root causes associated with the utilization of maternal health services in Myanmar—especially, the largely unheeded aspects at the intersection of modern and traditional practices to generate locally informed evidence.

## Figures and Tables

**Table 1:** Participants’ characteristics.

	Overall	Urban	Rural	
	(*n* = 258)	(*n* = 67)	(*n* = 191)	*p*-Value
	No. (%)	No. (%)	No. (%)	
Average age	31.7 ± 8.1	33.6 ± 8.7	31.0 ± 7.8	0.02
Marital status				0.33
Married	238 (93.3)	61 (91.0)	177 (94.1)	
Single	15 (5.9)	6 (9.0)	9 (4.8)	
Widowed	2 (0.8)	0 (0.0)	2 (1.1)	
Mother’s education				<0.01
None	4 (1.6)	0 (0.0)	4 (2.1)	
Nonstandard curriculum	3 (1.2)	1 (1.5)	2 (1.1)	
Primary school (grade 1–6)	68 (26.5)	14 (20.9)	54 (28.4)	
Lower secondary school (grade 7–9)	97 (37.7)	17 (25.4)	80 (42.1)	
Upper secondary school (grade 10–11)	36 (14.0)	10 (14.9)	26 (13.7)	
University	49 (19.1)	25 (37.3)	24 (12.7)	
Less than compulsory *	75 (29.2)	15 (22.4)	60 (31.6)	0.15
Household highest education				<0.01
None	7 (2.8)	0 (0.0)	7 (3.7)	
Nonstandard curriculum	3 (1.2)	0 (0.0)	3 (1.6)	
Primary school (grade 1–6)	38 (15.0)	4 (6.1)	34 (18.2)	
Lower secondary school (grade 7–9)	67 (26.5)	9 (13.6)	58 (31.0)	
Upper secondary school (grade 10–11)	69 (27.3)	19 (28.8)	50 (26.7)	
Vocational school	1 (0.4)	0 (0.0)	1 (0.5)	
University	68 (26.9)	34 (51.5)	34 (18.2)	
Less than compulsory	48 (19.0)	4 (6.1)	44 (23.5)	<0.01
Occupation				<0.01
Housewife	103 (42.6)	23 (35.9)	80 (44.9)	
Other	42 (17.4)	18 (28.1)	24 (13.5)	
Self-employed (exc. street vendor)	39 (16.1)	11 (17.2)	28 (15.7)	
Factory/manufacturing	17 (7.0)	1 (1.6)	16 (9.0)	
Street vendor	13 (5.4)	5 (7.8)	8 (4.5)	
Agriculture	12 (5.0)	0 (0.0)	12 (6.7)	
Government	9 (3.7)	6 (9.4)	3 (1.7)	
Unemployed	3 (1.2)	0 (0.0)	3 (1.7)	
Construction/other labor	2 (0.8)	0 (0.0)	2 (1.1)	
Teacher/professor	1 (0.4)	0 (0.0)	1 (0.6)	
Driver	1 (0.4)	0 (0.0)	1 (0.6)	
Household monthly income				
Less than $200 (median)	103 (45.6)	20 (44.4)	83 (45.9)	0.87
Below the poverty line	83 (39.1)	23 (4.8)	63 (35.4)	0.02

* Compulsory education in Myanmar includes primary and lower secondary school. Number of people who had less than compulsory education based on the number of people with up to primary education.

**Table 2:** Access and utilization of antenatal care services.

	Overall	Urban	Rural	
	(*n* = 258)	(*n* = 67)	(*n* = 191)	*p*-Value
	No. (%)	No. (%)	No. (%)	
Type of facility used most commonly for maternal health services				<0.01
Government clinic/hospital	118 (48.6)	18 (30.0)	100 (54.6)	
Private clinic/hospital	93 (38.3)	37 (61.7)	56 (30.6)	
Traditional birth attendant	16 (6.6)	0 (0.0)	16 (8.7)	
MMCWA	9 (3.7)	2 (3.3)	7 (3.8)	
Other	7 (2.9)	3 (5.0)	4 (2.2)	
Number of women who made less than recommended number of antenatal care visits				
1^st^ trimester	37 (16.7)	7 (14.9)	30 (17.2)	0.70
2^nd^ trimester	62 (26.8)	14 (25.0)	48 (27.4)	0.71
3^rd^ trimester	133 (58.3)	23 (39.0)	110 (65.1)	<0.01
Reasons for not utilizing antenatal care *				
Perceived as unnecessary	25 (36.8)	3 (12.5)	22 (50.0)	<0.01
Lack of money	12 (18.8)	6 (25.0)	6(15.0)	0.32
Lack of transportation	13 (21.3)	5 (20.8)	8 (21.6)	0.94
Distance to health facility	9 (14.3)	0 (0.0)	9 (23.1)	0.01
Other	2 (4.3)	0 (0.0)	2 (6.3)	1.00
Average number of antenatal care visits for women fulfilling the minimum requirement of eight visits				
1^st^ trimester (recommended one)	2.3 ± 2.3	2.7 ± 2.8	2.2 ± 2.1	0.13
2^nd^ trimester (recommended two)	3.6 ± 3.6	4.0 ± 3.7	3.5 ± 3.6	0.40
3^rd^ trimester (recommended five)	5.3 ± 4.6	7.3 ± 5.7	4.6 ± 4.1	<0.01
Minutes to health care facility	22 ± 18	18 ± 16	24 ± 18	0.03
Minutes to antenatal care facility	30 ± 29	30 ± 44	30 ± 23	0.99
Utilization of maternal health services				
Physical examination	194 (84.7)	51 (87.9)	143 (83.6)	0.43
Tetanus vaccine	189 (83.3)	41 (71.9)	148 (87.1)	<0.01
Blood tests	183 (82.4)	40 (72.7)	143 (85.6)	0.03
Nutritional supplements	178 (82.4)	42 (76.4)	136 (84.5)	0.17
Ultrasound examination	174 (77.0)	41 (71.9)	133 (78.7)	0.29
Gynecological examination	167 (76.3)	38 (70.4)	129 (78.2)	0.24
HIV/STD testing	166 (75.1)	35 (63.6)	131 (78.9)	0.02
Other	28 (21.4)	9 (34.6)	19 (18.1)	0.07
Pregnancy complications detected	15 (6.1)	7 (10.9)	8 (4.4)	0.06

* For those women that did not seek antenatal care.

**Table 3:** Women receiving essential antenatal care services by type of health care facility.

	Physical Exam	Gynecological Exam	USG	HIV/STD Test	Blood Test	Nutritional Supplements	Tetanus Vaccine
Gov. clinic/hospital	104 (93.7)	86 (84.3)	91 (85.8)	90 (84.9)	98 (91.6)	90 (90.0)	103 (93.6)
Private clinic/hospital	74 (87.1)	67 (79.8)	73 (83.9)	65 (77.4)	69 (84.1)	71 (86.6)	67 (78.8)
MMCWA	7 (87.5)	7 (87.5)	9 (100)	8 (100)	8 (100)	7 (87.5)	8 (100)
Traditional birth attendant	4 (26.7)	3 (21.4)	0 (0.0)	2 (15.4)	3 (23.1)	4 (26.7)	5 (35.7)
Other	5 (83.3)	3 (50.5)	1 (14.3)	1 (16.7)	4 (57.1)	6 (85.7)	5 (100)
Overall	194 (86.2)	166 (77.6)	174 (78.4)	166 (76.5)	182 (83.9)	178 (84.0)	188 (84.7)
*p*-value	<0.01	<0.01	<0.01	<0.01	<0.01	<0.01	<0.01
Conventional ^§^	185 (90.7)	160 (82.5)	173 (85.6)	163 (82.3)	175 (88.8)	168 (88.4)	178 (87.7)
Traditional birth attendant	4 (26.7)	3 (21.4)	0 (0.0)	2 (15.4)	3 (23.1)	4 (26.7)	5 (35.7)
*p*-value	<0.01	<0.01	<0.01	<0.01	<0.01	<0.01	<0.01

**^§^** Conventional includes governmental, private clinic/hospital, and Myanmar Maternal and Child Welfare Association (MMCWA).

**Table 4:** Labor and delivery.

	Overall	Urban	Rural	
	(*n* = 258)	(*n* = 67)	(*n* = 191)	*p*-Value
	No. (%)	No. (%)	No. (%)	
Delivered baby with a skilled birth attendant	234 (94.7)	60 (93.8)	174 (95.1)	0.68
Place of last delivery				<0.01
Health care facility	175 (72.9)	57 (93.4)	118 (65.9)	
Home	65 (27.1)	4 (6.6)	61 (34.1)	
Reason for not delivering at a health care facility				<0.01
Prefer home delivery	43 (81.1)	0 (0.0)	43 (84.3)	
Lack of money	5 (9.4)	1 (50.0)	4 (7.8)	
Distance to a health care facility	2 (3.8)	1 (50.0)	1 (2.0)	
Lack of transportation	2 (3.8)	0 (0.0)	2 (3.9)	
Other	1 (1.9)	0 (0.0)	1 (2.0)	
Method of last delivery				0.43
Vaginally	140 (97.9)	24 (100)	116 (97.5)	
C-section	3 (2.1)	0 (0.0)	3 (2.5)	
Experienced spontaneous abortion	7 (3.0)	0 (0.0)	7 (4.1)	0.11
Delivered a stillborn baby	3 (1.3)	0 (0.0)	3 (1.7)	0.30

**Table 5:** Utilization of postnatal care services.

	Overall	Urban	Rural	
	(*n* = 258)	(*n* = 67)	(*n* = 191)	*p*-Value
	No. (%)	No. (%)	No. (%)	
Received care within 24 h after birth	217 (88.9)	60 (98.4)	157 (85.8)	0.01
Number of postnatal care checkups	2.3 ± 2.6	2.6 ± 2.0	2.2 ± 2.8	0.21
Visited by a skilled birth attendant within one week	191 (77.6)	47 (73.4)	144 (79.1)	0.35
Utilized the following services within postnatal checkup				
Physical examination	185 (79.9)	48 (85.7)	137 (77.8)	0.20
Breastfeeding counseling	180 (78.6)	48 (76.2)	132 (79.5)	0.58
Child growth and weight monitoring	169 (74.1)	43 (68.3)	126 (76.4)	0.21
Newborn illness counseling	169 (74.1)	40 (67.8)	129 (76.3)	0.20
Nutritional supplements	146 (65.5)	30 (51.7)	116 (70.3)	0.01
New parents counseling	139 (64.1)	31 (51.7)	108 (68.8)	0.02
Family planning counseling	128 (58.4)	29 (49.2)	99 (61.9)	0.09
Postpartum depression counseling	123 (57.5)	26 (43.3)	97 (63.0)	0.01
Blood test for anemia	104 (47.9)	27 (44.3)	77 (49.4)	0.50

**Table 6:** Adjusted odds ratio (AOR) for determinants of maternal health services.

	AOR (95% CI)	*p*-Value
Not attending antenatal care		
Living in a rural setting	17.7 (1.72 – 183)	0.02
Less than primary education	14.7 (0.80 – 272.26)	0.07
Household income is at least $200	1.8 (0.52 – 6.19)	0.36
Home delivery		
Highest household education lower than compulsory education	3.3 (1.72 – 6.38)	< 0.01
Living in a rural setting	5.0 (1.66 – 14.96)	<0.01
Household income is at least $200	0.8 (0.43 – 1.52)	0.51
Not receiving postnatal care		
Home delivery	38.5 (9.17 – 167)	< 0.01
Highest household education lower than compulsory education	2.1 (0.70 – 6.21)	0.19
Household income is at least $200	1.0 (0.37 – 2.56)	0.98
